# 4,4′-Bis(dimethyl­amino)benzhydryl phenyl sulfone

**DOI:** 10.1107/S160053680904642X

**Published:** 2009-11-07

**Authors:** Mahiuddin Baidya, Herbert Mayr, Peter Mayer

**Affiliations:** a Ludwig-Maximilians-Universität, Department, Butenandtstrasse 5–13, 81377 München, Germany

## Abstract

In the title compound, C_23_H_26_N_2_O_2_S, the sulfur-bound phenyl group is aligned approximately parallel to one of the two rings of the benzhydryl group, making a dihedral angle of 1.15 (9)°. The other forms a dihedral angle of 59.20 (9)° with the phenyl group bound to the S atom. In the crystal, mol­ecules are linked into strands along [100] by weak C—H⋯O contacts. Weak C–H⋯π inter­actions are also observed.

## Related literature

For the history of the sulfone anion, see: Hinsberg (1897 ▶, 1917 ▶); Meek & Fowler (1968 ▶); Kobayashi & Toriyabe (1985 ▶); Veenstra & Zwanenburg (1975 ▶); Weber *et al.* (1985 ▶); Mayr *et al.* (2001 ▶, 2008 ▶). For a related structure, see: Li & Su (2005 ▶). For the graph-set analysis of hydrogen-bond networks, see: Bernstein *et al.* (1995 ▶); Etter *et al.* (1990 ▶).
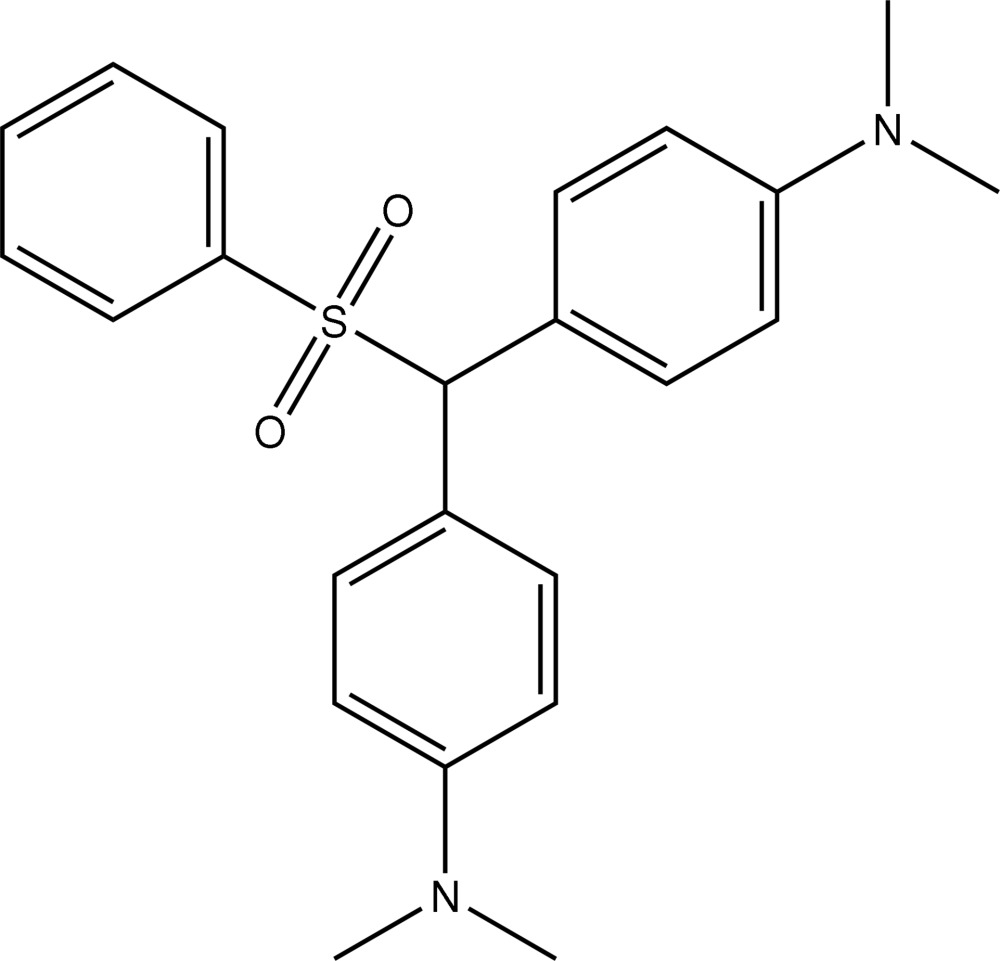


## Experimental

### 

#### Crystal data

C_23_H_26_N_2_O_2_S
*M* *_r_* = 394.53
Monoclinic, 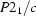

*a* = 5.9835 (2) Å
*b* = 16.6036 (5) Å
*c* = 20.8340 (6) Å
β = 98.150 (2)°
*V* = 2048.90 (11) Å^3^
*Z* = 4
Mo *K*α radiation
μ = 0.18 mm^−1^
*T* = 200 K
0.31 × 0.13 × 0.09 mm

#### Data collection

Nonius KappaCCD diffractometer
Absorption correction: none
13918 measured reflections
4468 independent reflections
3142 reflections with *I* > 2σ(*I*)
*R*_int_ = 0.050

#### Refinement

*R*[*F* ^2^ > 2σ(*F* ^2^)] = 0.043
*wR*(*F* ^2^) = 0.112
*S* = 1.03
4468 reflections
257 parameters
H-atom parameters constrained
Δρ_max_ = 0.19 e Å^−3^
Δρ_min_ = −0.33 e Å^−3^

### 

Data collection: *COLLECT* (Hooft, 2004 ▶); cell refinement: *SCALEPACK* (Otwinowski & Minor, 1997 ▶); data reduction: *DENZO* (Otwinowski & Minor, 1997 ▶) and *SCALEPACK*; program(s) used to solve structure: *SIR97* (Altomare *et al.*, 1999 ▶); program(s) used to refine structure: *SHELXL97* (Sheldrick, 2008 ▶); molecular graphics: *ORTEP-3* (Farrugia, 1997 ▶) and *Mercury* (Macrae *et al.*, 2006 ▶); software used to prepare material for publication: *PLATON* (Spek, 2009 ▶).

## Supplementary Material

Crystal structure: contains datablocks I, global. DOI: 10.1107/S160053680904642X/ng2679sup1.cif

Structure factors: contains datablocks I. DOI: 10.1107/S160053680904642X/ng2679Isup2.hkl

Additional supplementary materials:  crystallographic information; 3D view; checkCIF report

## Figures and Tables

**Table 1:** Hydrogen-bond geometry (Å, °)

*D*—H⋯*A*	*D*—H	H⋯*A*	*D*⋯*A*	*D*—H⋯*A*
C3—H3⋯O1^i^	0.95	2.56	3.469 (2)	160
C17—H17*C*⋯*Cg*1^ii^	0.98	2.80	3.673 (2)	148
C21—H21⋯*Cg*1^iii^	0.95	2.74	3.633 (2)	158
C22—H22⋯*Cg*2^iii^	0.95	2.78	3.435 (2)	127

Symmetry codes: (i) 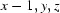; (ii) 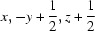; (iii) 
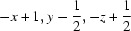
. *Cg*1 and *Cg*2 are the centroids of the C2–C7and C10–C15 rings, respectively.

**Fig. 1.**
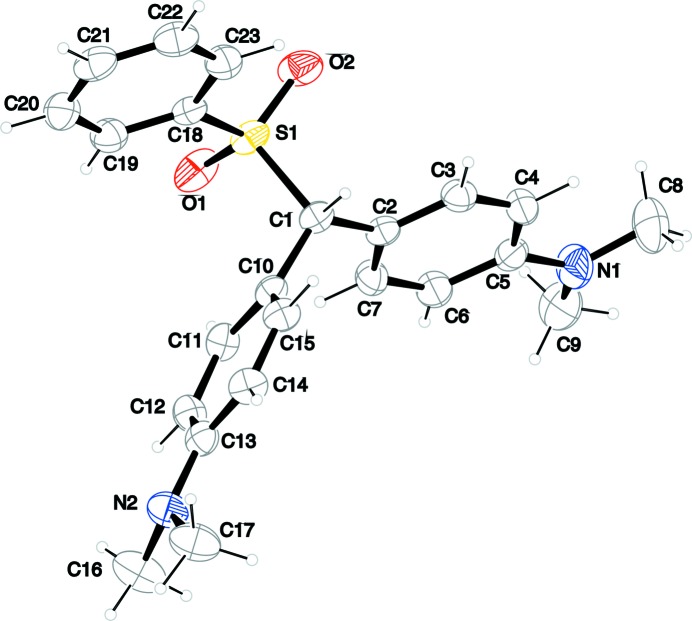
The molecular structure of the title compound, with atom labels and anisotropic displacement ellipsoids (drawn at 50% probability level) for non-H atoms.

**Fig. 2.**
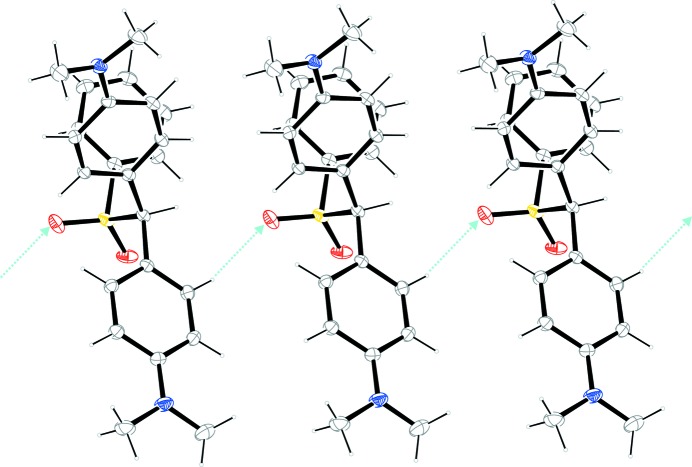
Weak intermolecular hydrogen bonds of the type C–H···O linking the title compound to strands along [100].

**Fig. 3.**
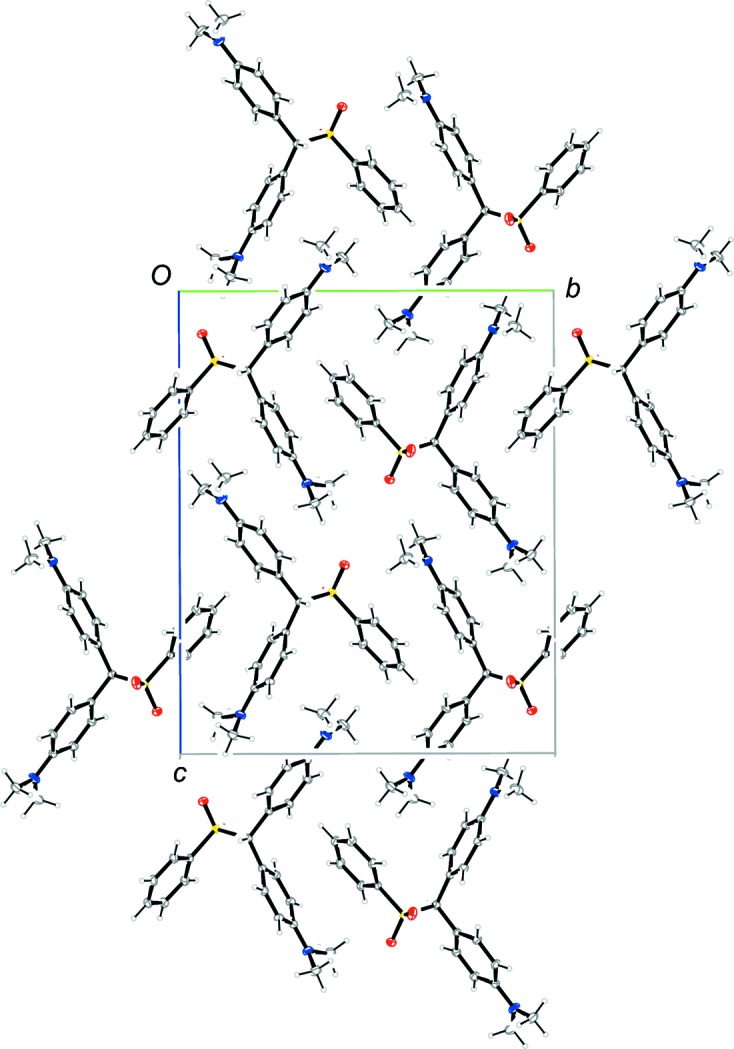
The packing of the title compound viewed along [100].

## supplementary crystallographic information

### Comment

During our studies on electrophile-nucleophile reactions [Mayr *et al.* (2001), Mayr *et al.* (2008)] we obtained a crystalline product from the reaction of sodium benzenesulfinate with 4,4'-bis(dimethylamino)benzhydrylium tetrafluoroborate in dimethyl sulfoxide which was characterized by X-ray crystallography to be 4,4'-bis(dimethylamino)benzhydryl phenyl sulfone.

The asymmetric unit of the title compound contains one complete molecule, which is shown in Figure 1. The sulfur-bound phenyl group is approximately parallel aligned to one of the two phenyl rings of the benzhydryl group with an dihedral angle of 1.15 (9)°. The other one forms a dihedral angle of 59.20 (9)° with the phenyl group bound to the sulfur atom.

The molecules are linked to strands along [100] by weak contacts of the type C–H···O (Fig. 2). Contacts of this type have been described for a structure of a related sulfone [Li *et al.* (2005)]. In terms of graph-set analysis [Bernstein *et al.* (1995), Etter *et al.* (1990)], the descriptor on the unitary level is C_1_^1^(6). Weak C–H···π interactions are also formed (see Table 1 for more details; *Cg*1 is the centre of gravity of the ring C2 to C7, *Cg*2 is the centre of gravity of the ring C10 to C15).

Parallel stacking of adjacent phenyl rings is not observed (Fig. 3). The dihedral angles exceed 46° in any cases.

### Experimental

The title compound was obtained by mixing sodium benzenesulfinate (36 mg, 0.22 mmol) and 4,4'-bis(dimethylamino)benzhydrylium tetrafluoroborate (74 mg, 0.22 mmol) in DMSO (8 ml) at room temperature. After 20 min of stirring, water (10 ml) was added, and the mixture was extracted with ethyl acetate. The organic layer was washed several times with brine and dried (MgSO_4_). After evaporation of volatile solvents under reduced pressure, 4,4'-bis(dimethylamino)benzhydryl phenyl sulfone (42 mg, 48%) was obtained as a colorless solid. A small amount of the solid was dissolved in ethyl acetate. The solvent was allowed to evaporate slowly at room temperature. After 2 days, crystals had formed that were suitable for X-ray analysis. mp 197 °C (mp 204 °C [Hinsberg (1897), Hinsberg (1917)].

### Refinement

All H atoms were found in difference maps. C-bound H atoms were positioned geometrically and treated as riding on their parent atoms [*U*_iso_(H) = 1.2*U*_eq_(C) for CH and *U*_iso_(H) = 1.5*U*_eq_(C) for CH_3_].

### Crystal data

**Table:**

C_23_H_26_N_2_O_2_S	*F*(000) = 840
*M**_r_* = 394.53	*D*_x_ = 1.279 (1) Mg m^−^^3^
Monoclinic, *P*2_1_/*c*	Mo *K*α radiation, λ = 0.71073 Å
Hall symbol: -P 2ybc	Cell parameters from 7329 reflections
*a* = 5.9835 (2) Å	θ = 3.1–27.1°
*b* = 16.6036 (5) Å	µ = 0.18 mm^−^^1^
*c* = 20.8340 (6) Å	*T* = 200 K
β = 98.150 (2)°	Rod, colourless
*V* = 2048.90 (11) Å^3^	0.31 × 0.13 × 0.09 mm
*Z* = 4	

### Data collection

**Table:**

Nonius KappaCCD diffractometer	3142 reflections with *I* > 2σ(*I*)
Radiation source: rotating anode	*R*_int_ = 0.050
MONTEL, graded multilayered X-ray optics	θ_max_ = 27.1°, θ_min_ = 3.4°
Detector resolution: 9 pixels mm^-1^	*h* = −7→7
CCD; rotation images; thick slices, phi/ω–scan	*k* = −21→20
13918 measured reflections	*l* = −25→26
4468 independent reflections	

### Refinement

**Table:**

Refinement on *F*^2^	Primary atom site location: structure-invariant direct methods
Least-squares matrix: full	Secondary atom site location: difference Fourier map
*R*[*F*^2^ > 2σ(*F*^2^)] = 0.043	Hydrogen site location: inferred from neighbouring sites
*wR*(*F*^2^) = 0.112	H-atom parameters constrained
*S* = 1.03	*w* = 1/[σ^2^(*F*_o_^2^) + (0.043*P*)^2^ + 0.5546*P*] where *P* = (*F*_o_^2^ + 2*F*_c_^2^)/3
4468 reflections	(Δ/σ)_max_ < 0.001
257 parameters	Δρ_max_ = 0.19 e Å^−^^3^
0 restraints	Δρ_min_ = −0.33 e Å^−^^3^

### Special details

**Table:**

Geometry. All e.s.d.'s (except the e.s.d. in the dihedral angle between two l.s. planes) are estimated using the full covariance matrix. The cell e.s.d.'s are taken into account individually in the estimation of e.s.d.'s in distances, angles and torsion angles; correlations between e.s.d.'s in cell parameters are only used when they are defined by crystal symmetry. An approximate (isotropic) treatment of cell e.s.d.'s is used for estimating e.s.d.'s involving l.s. planes.
Refinement. Refinement of *F*^2^ against ALL reflections. The weighted *R*-factor *wR* and goodness of fit *S* are based on *F*^2^, conventional *R*-factors *R* are based on *F*, with *F* set to zero for negative *F*^2^. The threshold expression of *F*^2^ > 2*σ*(*F*^2^) is used only for calculating *R*-factors(gt) *etc*. and is not relevant to the choice of reflections for refinement. *R*-factors based on *F*^2^ are statistically about twice as large as those based on *F*, and *R*-factors based on all data will be even larger.

### Fractional atomic coordinates and isotropic or equivalent isotropic displacement parameters (Å^2^)

**Table:**

	*x*	*y*	*z*	*U*_iso_*/*U*_eq_	
S1	0.74058 (8)	0.09486 (3)	0.15422 (2)	0.03368 (15)	
O1	0.9739 (2)	0.11658 (8)	0.15443 (7)	0.0452 (4)	
O2	0.6163 (2)	0.06134 (8)	0.09615 (6)	0.0460 (4)	
N1	0.3991 (3)	0.38915 (10)	−0.04522 (8)	0.0481 (4)	
N2	0.9032 (3)	0.34009 (9)	0.41719 (7)	0.0396 (4)	
C1	0.5830 (3)	0.18149 (10)	0.17654 (8)	0.0291 (4)	
H1	0.4310	0.1605	0.1829	0.035*	
C2	0.5424 (3)	0.23697 (10)	0.11785 (8)	0.0284 (4)	
C3	0.3353 (3)	0.23509 (11)	0.07836 (9)	0.0337 (4)	
H3	0.2231	0.1985	0.0883	0.040*	
C4	0.2872 (3)	0.28472 (11)	0.02511 (9)	0.0362 (4)	
H4	0.1426	0.2820	−0.0004	0.043*	
C5	0.4470 (3)	0.33879 (10)	0.00799 (9)	0.0334 (4)	
C6	0.6582 (3)	0.33989 (11)	0.04706 (9)	0.0369 (4)	
H6	0.7721	0.3755	0.0367	0.044*	
C7	0.7035 (3)	0.28988 (10)	0.10057 (9)	0.0343 (4)	
H7	0.8482	0.2918	0.1260	0.041*	
C8	0.1893 (4)	0.38272 (16)	−0.08687 (12)	0.0651 (7)	
H8A	0.1666	0.3269	−0.1017	0.098*	
H8B	0.1908	0.4182	−0.1244	0.098*	
H8C	0.0661	0.3986	−0.0631	0.098*	
C9	0.5726 (4)	0.43670 (14)	−0.06787 (11)	0.0557 (6)	
H9A	0.6485	0.4694	−0.0321	0.084*	
H9B	0.5049	0.4721	−0.1030	0.084*	
H9C	0.6828	0.4011	−0.0840	0.084*	
C10	0.6815 (3)	0.21879 (10)	0.24073 (8)	0.0288 (4)	
C11	0.8948 (3)	0.25420 (11)	0.25295 (9)	0.0326 (4)	
H11	0.9933	0.2505	0.2211	0.039*	
C12	0.9664 (3)	0.29446 (11)	0.31010 (8)	0.0334 (4)	
H12	1.1117	0.3187	0.3162	0.040*	
C13	0.8299 (3)	0.30059 (10)	0.35951 (8)	0.0322 (4)	
C14	0.6190 (3)	0.26226 (11)	0.34831 (8)	0.0345 (4)	
H14	0.5230	0.2633	0.3809	0.041*	
C15	0.5488 (3)	0.22278 (10)	0.29024 (9)	0.0325 (4)	
H15	0.4048	0.1976	0.2841	0.039*	
C16	1.0909 (4)	0.39582 (15)	0.42066 (11)	0.0586 (6)	
H16A	1.2192	0.3692	0.4051	0.088*	
H16B	1.1346	0.4130	0.4657	0.088*	
H16C	1.0459	0.4429	0.3936	0.088*	
C17	0.7429 (4)	0.35926 (14)	0.46134 (10)	0.0514 (6)	
H17A	0.6346	0.3993	0.4412	0.077*	
H17B	0.8241	0.3811	0.5018	0.077*	
H17C	0.6621	0.3103	0.4708	0.077*	
C18	0.7267 (3)	0.02533 (10)	0.21776 (8)	0.0290 (4)	
C19	0.9132 (3)	0.01217 (11)	0.26374 (9)	0.0366 (4)	
H19	1.0483	0.0418	0.2624	0.044*	
C20	0.9012 (4)	−0.04461 (11)	0.31178 (10)	0.0425 (5)	
H20	1.0293	−0.0545	0.3433	0.051*	
C21	0.7036 (4)	−0.08691 (11)	0.31415 (9)	0.0412 (5)	
H21	0.6956	−0.1252	0.3476	0.049*	
C22	0.5181 (4)	−0.07361 (11)	0.26796 (10)	0.0423 (5)	
H22	0.3828	−0.1030	0.2696	0.051*	
C23	0.5285 (3)	−0.01744 (11)	0.21925 (9)	0.0373 (4)	
H23	0.4013	−0.0084	0.1873	0.045*	

### Atomic displacement parameters (Å^2^)

**Table:**

	*U*^11^	*U*^22^	*U*^33^	*U*^12^	*U*^13^	*U*^23^
S1	0.0452 (3)	0.0261 (2)	0.0328 (3)	0.00373 (19)	0.0159 (2)	0.00259 (18)
O1	0.0450 (8)	0.0352 (7)	0.0617 (9)	0.0052 (6)	0.0296 (7)	0.0088 (6)
O2	0.0762 (10)	0.0358 (7)	0.0268 (7)	0.0050 (7)	0.0096 (7)	−0.0029 (6)
N1	0.0550 (11)	0.0468 (10)	0.0399 (10)	0.0014 (8)	−0.0024 (8)	0.0149 (8)
N2	0.0448 (10)	0.0429 (9)	0.0320 (9)	−0.0050 (8)	0.0083 (7)	−0.0045 (7)
C1	0.0330 (10)	0.0264 (9)	0.0297 (9)	0.0013 (7)	0.0107 (8)	0.0004 (7)
C2	0.0333 (10)	0.0247 (9)	0.0285 (9)	0.0011 (7)	0.0090 (8)	−0.0016 (7)
C3	0.0326 (10)	0.0307 (9)	0.0382 (10)	−0.0040 (8)	0.0062 (8)	−0.0015 (8)
C4	0.0320 (10)	0.0399 (10)	0.0351 (10)	0.0014 (8)	−0.0008 (8)	−0.0011 (8)
C5	0.0414 (11)	0.0282 (9)	0.0306 (10)	0.0062 (8)	0.0048 (8)	0.0001 (7)
C6	0.0386 (11)	0.0327 (10)	0.0389 (11)	−0.0048 (8)	0.0041 (9)	0.0064 (8)
C7	0.0336 (10)	0.0334 (10)	0.0347 (10)	−0.0016 (8)	0.0008 (8)	0.0045 (8)
C8	0.0605 (15)	0.0772 (17)	0.0527 (15)	0.0080 (13)	−0.0092 (12)	0.0214 (13)
C9	0.0688 (15)	0.0517 (13)	0.0464 (13)	0.0004 (12)	0.0075 (11)	0.0193 (11)
C10	0.0334 (10)	0.0250 (9)	0.0287 (9)	0.0031 (7)	0.0070 (8)	0.0033 (7)
C11	0.0316 (10)	0.0373 (10)	0.0304 (10)	0.0046 (8)	0.0094 (8)	0.0036 (8)
C12	0.0297 (9)	0.0379 (10)	0.0327 (10)	−0.0022 (8)	0.0041 (8)	0.0037 (8)
C13	0.0385 (10)	0.0307 (9)	0.0275 (9)	0.0025 (8)	0.0051 (8)	0.0050 (7)
C14	0.0387 (11)	0.0372 (10)	0.0303 (10)	−0.0012 (8)	0.0144 (8)	0.0005 (8)
C15	0.0346 (10)	0.0291 (9)	0.0358 (10)	−0.0029 (8)	0.0116 (8)	0.0012 (8)
C16	0.0574 (14)	0.0714 (16)	0.0481 (13)	−0.0245 (12)	0.0108 (11)	−0.0139 (12)
C17	0.0615 (14)	0.0580 (13)	0.0373 (12)	−0.0138 (11)	0.0163 (10)	−0.0125 (10)
C18	0.0384 (10)	0.0210 (8)	0.0287 (9)	0.0003 (7)	0.0085 (8)	−0.0014 (7)
C19	0.0377 (10)	0.0316 (10)	0.0407 (11)	−0.0037 (8)	0.0063 (9)	−0.0001 (8)
C20	0.0516 (13)	0.0361 (10)	0.0377 (11)	0.0027 (9)	−0.0006 (9)	0.0041 (8)
C21	0.0629 (14)	0.0270 (10)	0.0361 (11)	−0.0007 (9)	0.0149 (10)	0.0048 (8)
C22	0.0505 (12)	0.0308 (10)	0.0483 (12)	−0.0095 (9)	0.0165 (10)	0.0007 (9)
C23	0.0394 (11)	0.0322 (10)	0.0401 (11)	−0.0030 (8)	0.0052 (9)	0.0009 (8)

### Geometric parameters (Å, °)

**Table:**

S1—O2	1.4391 (13)	C9—H9C	0.9800
S1—O1	1.4413 (14)	C10—C15	1.390 (2)
S1—C18	1.7672 (17)	C10—C11	1.396 (2)
S1—C1	1.8163 (17)	C11—C12	1.380 (2)
N1—C5	1.386 (2)	C11—H11	0.9500
N1—C8	1.425 (3)	C12—C13	1.405 (3)
N1—C9	1.436 (3)	C12—H12	0.9500
N2—C13	1.385 (2)	C13—C14	1.403 (3)
N2—C16	1.449 (3)	C14—C15	1.388 (2)
N2—C17	1.455 (3)	C14—H14	0.9500
C1—C10	1.515 (2)	C15—H15	0.9500
C1—C2	1.523 (2)	C16—H16A	0.9800
C1—H1	1.0000	C16—H16B	0.9800
C2—C3	1.387 (2)	C16—H16C	0.9800
C2—C7	1.389 (2)	C17—H17A	0.9800
C3—C4	1.379 (3)	C17—H17B	0.9800
C3—H3	0.9500	C17—H17C	0.9800
C4—C5	1.394 (3)	C18—C19	1.381 (2)
C4—H4	0.9500	C18—C23	1.386 (2)
C5—C6	1.403 (3)	C19—C20	1.384 (3)
C6—C7	1.386 (2)	C19—H19	0.9500
C6—H6	0.9500	C20—C21	1.382 (3)
C7—H7	0.9500	C20—H20	0.9500
C8—H8A	0.9800	C21—C22	1.380 (3)
C8—H8B	0.9800	C21—H21	0.9500
C8—H8C	0.9800	C22—C23	1.386 (3)
C9—H9A	0.9800	C22—H22	0.9500
C9—H9B	0.9800	C23—H23	0.9500
			
O2—S1—O1	118.94 (9)	C15—C10—C11	116.64 (16)
O2—S1—C18	107.68 (8)	C15—C10—C1	118.88 (15)
O1—S1—C18	108.04 (8)	C11—C10—C1	124.38 (15)
O2—S1—C1	107.24 (8)	C12—C11—C10	121.71 (17)
O1—S1—C1	109.81 (8)	C12—C11—H11	119.1
C18—S1—C1	104.14 (8)	C10—C11—H11	119.1
C5—N1—C8	120.42 (18)	C11—C12—C13	121.72 (17)
C5—N1—C9	121.15 (17)	C11—C12—H12	119.1
C8—N1—C9	117.16 (17)	C13—C12—H12	119.1
C13—N2—C16	119.58 (16)	N2—C13—C14	121.74 (16)
C13—N2—C17	119.74 (16)	N2—C13—C12	121.64 (17)
C16—N2—C17	113.86 (16)	C14—C13—C12	116.59 (16)
C10—C1—C2	117.33 (14)	C15—C14—C13	120.88 (16)
C10—C1—S1	113.48 (12)	C15—C14—H14	119.6
C2—C1—S1	107.63 (11)	C13—C14—H14	119.6
C10—C1—H1	105.8	C14—C15—C10	122.38 (17)
C2—C1—H1	105.8	C14—C15—H15	118.8
S1—C1—H1	105.8	C10—C15—H15	118.8
C3—C2—C7	117.06 (16)	N2—C16—H16A	109.5
C3—C2—C1	119.33 (15)	N2—C16—H16B	109.5
C7—C2—C1	123.61 (15)	H16A—C16—H16B	109.5
C4—C3—C2	122.04 (17)	N2—C16—H16C	109.5
C4—C3—H3	119.0	H16A—C16—H16C	109.5
C2—C3—H3	119.0	H16B—C16—H16C	109.5
C3—C4—C5	121.21 (16)	N2—C17—H17A	109.5
C3—C4—H4	119.4	N2—C17—H17B	109.5
C5—C4—H4	119.4	H17A—C17—H17B	109.5
N1—C5—C4	121.43 (17)	N2—C17—H17C	109.5
N1—C5—C6	121.57 (17)	H17A—C17—H17C	109.5
C4—C5—C6	116.99 (16)	H17B—C17—H17C	109.5
C7—C6—C5	121.09 (17)	C19—C18—C23	120.87 (16)
C7—C6—H6	119.5	C19—C18—S1	120.24 (14)
C5—C6—H6	119.5	C23—C18—S1	118.85 (13)
C6—C7—C2	121.59 (17)	C18—C19—C20	119.31 (18)
C6—C7—H7	119.2	C18—C19—H19	120.3
C2—C7—H7	119.2	C20—C19—H19	120.3
N1—C8—H8A	109.5	C21—C20—C19	120.29 (18)
N1—C8—H8B	109.5	C21—C20—H20	119.9
H8A—C8—H8B	109.5	C19—C20—H20	119.9
N1—C8—H8C	109.5	C22—C21—C20	120.09 (18)
H8A—C8—H8C	109.5	C22—C21—H21	120.0
H8B—C8—H8C	109.5	C20—C21—H21	120.0
N1—C9—H9A	109.5	C21—C22—C23	120.20 (18)
N1—C9—H9B	109.5	C21—C22—H22	119.9
H9A—C9—H9B	109.5	C23—C22—H22	119.9
N1—C9—H9C	109.5	C22—C23—C18	119.23 (17)
H9A—C9—H9C	109.5	C22—C23—H23	120.4
H9B—C9—H9C	109.5	C18—C23—H23	120.4
			
O2—S1—C1—C10	174.47 (12)	C15—C10—C11—C12	−3.0 (3)
O1—S1—C1—C10	−54.96 (14)	C1—C10—C11—C12	173.26 (16)
C18—S1—C1—C10	60.52 (14)	C10—C11—C12—C13	1.2 (3)
O2—S1—C1—C2	−53.97 (13)	C16—N2—C13—C14	−163.95 (19)
O1—S1—C1—C2	76.60 (13)	C17—N2—C13—C14	−14.6 (3)
C18—S1—C1—C2	−167.92 (11)	C16—N2—C13—C12	18.3 (3)
C10—C1—C2—C3	−131.26 (17)	C17—N2—C13—C12	167.64 (18)
S1—C1—C2—C3	99.33 (16)	C11—C12—C13—N2	179.12 (16)
C10—C1—C2—C7	49.4 (2)	C11—C12—C13—C14	1.3 (3)
S1—C1—C2—C7	−80.00 (19)	N2—C13—C14—C15	−179.82 (16)
C7—C2—C3—C4	−1.6 (3)	C12—C13—C14—C15	−2.0 (3)
C1—C2—C3—C4	179.00 (16)	C13—C14—C15—C10	0.2 (3)
C2—C3—C4—C5	0.8 (3)	C11—C10—C15—C14	2.3 (3)
C8—N1—C5—C4	−4.7 (3)	C1—C10—C15—C14	−174.19 (16)
C9—N1—C5—C4	−171.44 (19)	O2—S1—C18—C19	140.96 (15)
C8—N1—C5—C6	175.4 (2)	O1—S1—C18—C19	11.31 (17)
C9—N1—C5—C6	8.6 (3)	C1—S1—C18—C19	−105.40 (15)
C3—C4—C5—N1	−179.57 (17)	O2—S1—C18—C23	−36.90 (16)
C3—C4—C5—C6	0.4 (3)	O1—S1—C18—C23	−166.54 (14)
N1—C5—C6—C7	179.29 (17)	C1—S1—C18—C23	76.74 (15)
C4—C5—C6—C7	−0.7 (3)	C23—C18—C19—C20	0.1 (3)
C5—C6—C7—C2	−0.2 (3)	S1—C18—C19—C20	−177.73 (14)
C3—C2—C7—C6	1.3 (3)	C18—C19—C20—C21	−0.8 (3)
C1—C2—C7—C6	−179.32 (16)	C19—C20—C21—C22	1.0 (3)
C2—C1—C10—C15	112.91 (18)	C20—C21—C22—C23	−0.4 (3)
S1—C1—C10—C15	−120.48 (15)	C21—C22—C23—C18	−0.3 (3)
C2—C1—C10—C11	−63.2 (2)	C19—C18—C23—C22	0.5 (3)
S1—C1—C10—C11	63.4 (2)	S1—C18—C23—C22	178.34 (14)

### Hydrogen-bond geometry (Å, °)

**Table:**

*D*—H···*A*	*D*—H	H···*A*	*D*···*A*	*D*—H···*A*
C3—H3···O1^i^	0.95	2.56	3.469 (2)	160
C17—H17C···Cg1^ii^	0.98	2.80	3.673 (2)	148
C21—H21···Cg1^iii^	0.95	2.74	3.633 (2)	158
C22—H22···Cg2^iii^	0.95	2.78	3.435 (2)	127

Symmetry codes: (i) *x*−1, *y*, *z*; (ii) *x*, −*y*+1/2, *z*+1/2; (iii) −*x*+1, *y*−1/2, −*z*+1/2.
